# Effects of Pulmonary Rehabilitation on Respiratory Function and Thickness of the Diaphragm in Patients with Post-COVID-19 Syndrome: A Randomized Clinical Trial

**DOI:** 10.3390/jcm13020425

**Published:** 2024-01-12

**Authors:** Katarzyna Anna Pietranis, Wiktoria Maria Izdebska, Anna Kuryliszyn-Moskal, Agnieszka Dakowicz, Mariusz Ciołkiewicz, Katarzyna Kaniewska, Zofia Dzięcioł-Anikiej, Mariusz Wojciuk

**Affiliations:** 1 Department of Rehabilitation, Medical University of Bialystok, 24A M. Skłodowskiej-Curie St., 15-276 Bialystok, Poland; katarzyna.pietranis@sd.umb.edu.pl (K.A.P.); anna.kuryliszyn-moskal@umb.edu.pl (A.K.-M.); agnieszka.dakowicz@umb.edu.pl (A.D.); mariusz.ciolkiewicz@umb.edu.pl (M.C.); katarzyna.kaniewska@umb.edu.pl (K.K.); mariusz.wojciuk@umb.edu.pl (M.W.); 2 Department of Gastroenterology and Internal Medicine, Medical University of Bialystok, 24A M. Skłodowskiej-Curie St., 15-276 Bialystok, Poland; izdebskawiktoria@gmail.com

**Keywords:** COVID-19, post-COVID-19, long COVID-19, pulmonary rehabilitation, respiratory muscles training

## Abstract

Background: Rehabilitation is an effective method for improving the overall health of patients who have experienced the long-term effects of COVID-19. Methods: The double-blind, randomized prospective study assessed the effectiveness of a 6-week rehabilitation program among post-COVID-19 patients. A total of 59 patients under treatment following COVID-19 were randomly divided into two groups. Both groups completed the same six-week comprehensive exercise training program supported by a respiratory muscle trainer (Threshold IMT) during out-patient sessions. The control group performed placebo IMT. Respiratory muscle strength, chest wall expansion, spirometry, and diaphragm ultrasonography measurements were taken before and after the six weeks. Results: The applied rehabilitation program improved respiratory muscle strength in both the study and control groups (*p* < 0.001). There was a significant chest circumference increase in the study group (*p* < 0.001). Spirometric parameters improved in both groups, with the study group showing a greater improvement: 8.02% in FEV1 (*p* < 0.001), 13.24% in FVC EX (*p* < 0.001) and 9.67% in PEF (*p* < 0.001). Rehabilitation also increased diaphragm thickness during maximum inhalation in both groups. Conclusions: Based on the study findings, the specialized outpatient rehabilitation program developed for post-COVID-19 patients has proven to be effective and safe.

## 1. Introduction

At the end of 2019, the first official case of infection with the new betacoronavirus SARS-CoV-2 was confirmed in the Chinese province of Hubei in the city of Wuhan [1]. SARS-CoV-2 (severe acute respiratory syndrome coronavirus 2) is a virus that causes severe acute respiratory syndrome—a disease called COVID-19. At the point of the widespread transmission of the virus, the World Health Organization (WHO) recognized COVID-19 as a pandemic on 11 March 2020 [2]. Presently, more than 772 million cases of infections have been confirmed worldwide with more than 6.9 million deaths as a result of COVID-19 [3].

COVID-19 is a highly contagious disease of the respiratory system leading to physical and mental dysfunctions [4]. Patients with this disease usually express a restrictive pulmonary pattern with additional impaired diffusion capacity. Fever, dry cough, dyspnea, myalgia, fatigue, normal or decreased leukocyte counts, and radiographic evidence of pneumonia are the most common symptoms of patients infected with COVID-19. Additional symptoms such as radiological ground-glass lung changes, lymphopenia, and thrombocytopenia symptoms have been reported [5,6,7]. In outpatient-treated patients, the most commonly observed symptoms include chronic cough, breathlessness, chest tightness, cognitive dysfunction, and extreme fatigue [8,9,10,11]. Many patients suffer from problems such as dizziness, sensations of swaying or spinning, and imbalance [12]. Prolonged hospitalization and extended periods of lying down contribute to muscle weakness, including respiratory muscles. This, in turn, can impede the transition to an upright position, potentially delaying full recovery and independence. Therefore, many individuals recovering from COVID-19 face difficulties in their daily activities, such as mobility, balance, and coordination. This functional impairment is apparent after both mild and severe cases. This indicates the necessity for specific rehabilitation strategies. Reduced exercise tolerance and heightened fatigue during physical activities further emphasize the crucial role of individualized exercise programs in the rehabilitation process. The broad spectrum of the virus’s activity, along with a substantial number of severe cases necessitating hospitalization, prompted clinicians and researchers from around the world to focus on severe cases when designing clinical guidelines, aiming to reduce the burden on hospital facilities [13,14]. The acute phase and long-term symptoms after discharge of the disease have been widely described [8,15,16]. An often observed, long-term impairment occurs in patients following SARS-CoV-2 infection, yet the pathophysiological processes and mechanisms underlying the condition known as post-COVID-19 syndrome remain unfathomable. Long COVID-19 is a pathology associated with persistent physical, medical, and subjective consequences continuing for at least 12 weeks after SARS-CoV-2 infection, persisting and reappearing for several weeks. Almost half of COVID-19 survivors, regardless of hospitalization status, develop at least one persistent symptom [17,18,19]. The aftermath of long COVID-19 includes persistent immunosuppression, diabetes, and manifestations of pulmonary, cardiac, and vascular fibrosis [20,21,22,23,24], significantly limiting functional efficiency, the ability to fulfill social roles, and the return to work. This induces the need for interventions that will best contribute to improving the quality of life, reducing the frequency of hospitalizations, and mitigating the demand for specialized medical services, which can otherwise increase healthcare expenditures and strain available resources [25,26]. Unfortunately, there is currently no specific pharmaceutical treatment designated to alleviate the symptoms of long COVID-19 [23,27]. However, it is known that the most effective approach to treating and alleviating these symptoms involves individualized and multidisciplinary rehabilitation [28,29,30,31,32,33].

Moreover, Daynes et al. stated directly a need for rehabilitation after COVID-19 infection in patients all over the world [34]. This underscores the pressing need to improve the lung and physical capacities of long COVID-19 patients. Identifying key rehabilitation aspects is vital for designing effective physiotherapy programs. Exploring interventions to enhance respiratory function could significantly elevate the quality of post-COVID-19 rehabilitation.

Unfortunately, not many studies have been published on the subject of post-COVID-19 rehabilitation. The current database of programs, encompassing pulmonary, aerobic, and resistance exercises and showcasing enhancements in both cardiorespiratory and musculoskeletal fitness, is limited. Therefore, additional research is crucial to identify and assess the effectiveness of both individual physiotherapeutic interventions and structured, comprehensive rehabilitation programs.

The main aim of this study is to assess the effectiveness of the original rehabilitation program on the functional parameters of the respiratory system, including the strength of the respiratory muscles and the diaphragm thickness, to assess its function in patients after COVID-19. 

## 2. Materials and Methods

### 2.1. Trial Design

The conducted interventional, double-blind, prospective randomized controlled trial (RCT) consisted of two parallel arms with a random allocation of participants into groups at a 1:1 ratio. Despite the assumed allocation of respondents to groups in a 1:1 ratio, the final distribution of participants was closer to a 2:1 ratio. The analyzed data do not constitute the entire database but are part of an open project. Nevertheless, we decided to present promising preliminary results. The protocol was not modified after patients began qualifying for the study. Due to the insufficient data on the effectiveness of IMT-supported exercise training (ET), a minimum group size of 60 participants was arbitrarily assumed.

The presented data are part of a project conducted since 8 July 2022 at the Department of Rehabilitation of the Medical University of Bialystok, Poland (ClinicalTrials.gov ID: NCT05449379) encompassing data acquired from August 2022 to June 2023. The study was conducted in accordance with the Declaration of Helsinki Principles for Medical Research Involving Human Subjects and was approved by the Bioethical Committee of the Medical University of Bialystok, Poland (APK.002.51.2022). Prior to the commencement of the study, we obtained informed consent from each participant, both verbally and in writing. We ensured that all subjects were fully informed about the study’s objectives, procedures, as well as any potential risks or discomfort associated with their participation.

### 2.2. Participants

The primary inclusion criterion was a confirmed history of COVID-19, based on the results of reverse-transcription polymerase chain reaction (RT-PCR) tests conducted on nasopharyngeal swabs, as reported by the patients. The eligibility criteria for patients included the presence of systemic post-COVID-19 complications assessed on a scale of 0 to 4 (with a score of 1–4) based on the Post-COVID-19 Functional Status (PCFS) scale; dyspnea with a score of ≥1 on the modified Medical Research Council (mMRC) dyspnea scale (0–4). Participation in the study was restricted to patients who had provided informed consent.

The study’s exclusion criteria included individuals under 18 years of age and those who did not provide consent, as well as those who had participated in another rehabilitation program. Patients with chronic musculoskeletal conditions, referred pain to internal organs, or certain medical conditions (e.g., pregnancy, tuberculosis, deep vein thrombosis, cancer, and demyelinating diseases) were excluded from the study. Additionally, individuals unable to obtain valid results or perform pulmonary function tests due to health issues or contraindications were not included.

### 2.3. Interventions

Both groups underwent identical physiotherapeutic interventions consisting of individual exercise training, which included (1) aerobic training on a cycle ergometer (interval training starting with a cycle of 4 min/2 min in the first week, lasting from 15 to 20 min, with a progressive time progression to 30–45 min and shortening the rest phase with improving performance in the sixth week; intensity maintained in the range of 45% to 55% to 70–80% of the maximum heart rate achieved during an exercise stress test), (2) respiratory training to restore proper breathing patterns and enhance chest mobility (10–15 min), (3) resistance training targeting major muscle groups (e.g., weight training on machines, free weight, elastic resistance bands; exercise intensity adapted to the individual’s capabilities, with a progression of load and repetitions of 8–10–12 in successive weeks; duration of 10–15 min), (4) overall fitness exercises, and stretching routines (e.g., post-isometric relaxation; 10–15 min). The study group (IMT group) underwent additional (5) resistance training using a respiratory muscle trainer (Philips Respironics Threshold IMT) during each ambulatory training session (6 sets of 6 maximally deep breaths exceeding the threshold value of pressure from 45–55% to 70–80% of PImax; duration of 15–20 min).

The control group (placebo group) performed the described training element in a placebo form without the mechanism providing resistance found in the Philips Respironics Threshold IMT device. Additionally, both groups received (6) education on managing bothersome symptoms and engaging in safe physical activity. Load progression was applied in accordance with Table 1. The modified Borg rating of perceived exertion (RPE) scale was used to determine exercise intensity at the level of 5–6/10. The intervention was conducted over six weeks, with sessions held three times a week, each lasting 1–1.5 h. To ensure a high level of adherence, in case a training session was missed, the patient could complete or make up for the training on the following day. The training was conducted in an outpatient setting under the one-to-one supervision of an experienced physiotherapist and supplemented with individually prescribed home exercises. 

### 2.4. Randomisation

The study was conducted in a double-blind manner. The patients were not aware of the group assignment (unaware of the existence of the intervention and control groups, as well as the differences in training—resistance or no resistance in the Philips Respironics Threshold IMT device), and the researchers assessing the functional status in the subsequent stages of the study did not conduct rehabilitation and were not aware of the patients’ group assignment. The participants were randomly allocated to one of two groups using Research Randomizer (Version 4.0) [35] by an independent researcher who was not involved in patient qualification, data collection, or study result analysis.

Qualification and data collection were conducted by a researcher who did not administer training and remained unaware of participants’ group assignments throughout the study. The investigator responsible for administering ET was not engaged in eligibility determination, data collection, or result analysis. All investigators, staff, and participants were kept masked to outcome measurements and trial results.

The physiotherapist responsible for conducting ET received specific, individualized guidelines concerning each patient’s training parameters. Additionally, study participants from both groups received training in the principles of safe physical activity and how to manage any troublesome symptoms.

### 2.5. Measures

The primary outcome in relation to the effectiveness of post-COVID-19 rehabilitation was maximum inspiratory pressure (PImax). In addition to this parameter we also evaluated maximum expiratory pressure (PEmax). We utilized measurements of chest mobility, spirometric parameters, and ultrasound measurements of the diaphragm as secondary outcomes.

We conducted the assessments in the following sequence: respiratory pressure measurements, diaphragm ultrasonography, spirometry, and chest wall mobility. All data were collected during morning hours.

Maximum inspiratory and expiratory pressure were assessed during forced inspiration and expiration, using the MicroRPM respiratory pressure meter (Micromedical Ltd., Rochester, Kent, UK). The measurement was performed in accordance with the procedure described by Wojciuk et al. [36] and following the recommendations of the American Thoracic Society and the European Respiratory Society guidelines [37,38,39]. The result consisted of the measurements of the maximum average inspiratory and expiratory pressure sustained during the first second of the test, both expressed in centimeters of water (cmH_2_O). The best result out of three repeated attempts was recorded for each measurement.

Chest expansion was assessed using a measuring tape at two distinct rib cage levels based on anatomical landmarks: xiphoid process and 10th rib. Clear instructions were provided to the participants, and a demonstration was conducted to ensure their full comprehension of the procedure. Chest circumference measurements were taken in a sitting position during both deep inhalation and exhalation. The examiner performed two consecutive measurements: first, an assessment of upper (xiphoid process level) and then an evaluation of lower (10th rib level) chest expansion. The measurements were presented in centimeters. Both values were calculated by subtracting the inspiratory diameter from the expiratory diameter, with reference to the designated anatomical landmarks.

Pulmonary function tests (PFT) were performed with a Lungtest 1000 (MES, Poland) according to the American Thoracic Society and the European Respiratory Society guidelines [37,38,39]. The recorded parameters were forced expiratory volume in 1 s (FEV1), forced expiratory vital capacity (FVC EX), forced inspiratory vital capacity (FVC IN), peak expiratory flow (PEF), maximum vital capacity (VC MAX), maximal expiratory flow in range 75–25% of FVC (MEF25, MEF50, MEF75), FEV1/FVC ratio, and FEV1/VC MAX ratio. Three to eight spirometric measurements were conducted to obtain a minimum of three reproducible readings. ERS norm values for patients aged 71 and above were calculated through approximation. Norms for the Caucasian race were applied, taking into account the subject’s gender, height, body mass, and age. The analysis involved utilizing the results expressed as a percentage of the predicted value, calculated using the actual/predicted equation.

The ultrasonographic measurement of diaphragm thickness was performed with the patient in the supine position and their upper limbs elevated above the head. Right and left hemidiaphragms were sonographically visualized via an intercostal approach. B-mode was used and a linear transducer L12-4 with a 42 Hz probe with the internal MSK program of the Philips ClearVue 550 (Philips, Netherlands) was utilized. Ultrasonography was performed in accordance with the literature [40]. An intercostal approach was used between the anterior–axillary and mid-axillary lines at the 8th and 9th ribs, or 9th and 10th ribs, with the probe marker pointing toward the head [40,41], and 3 to 5 images of the diaphragm’s respiratory phases during normal and forced breathing were taken. The diaphragm was measured on each respiratory phase [42]. Diaphragm function was assessed using the diaphragm thickening fraction (DTF), which was calculated using the formula [41,43]:

$$DTF=\frac{diaphragmatic\;thickness\;at\;end-inspiration/diaphragmatic\;thickness\;at\;end-expiration}{diaphragmatic\;thickness\;at\;end-expiration}$$

No changes or modifications to trial outcomes were performed after the trial commenced.

### 2.6. Statistical Methods

Statistical methods used to compare groups and outcomes included descriptive statistics (mean, median, interquartile range, normative description), Wilcoxon and U Mann–Whitney tests, as well as Spearman’s correlations tests using the Statistica (version 13.3) program. We assessed the data for statistical significance, which was determined by *p* < 0.05. Additionally, we performed correlations between all spirometry parameters in patients after pulmonary rehabilitation. In order to do so, we performed Spearman’s correlation test with the cut-off point for statistical significance being *p* < 0.05.

## 3. Results

**Figure 1:**
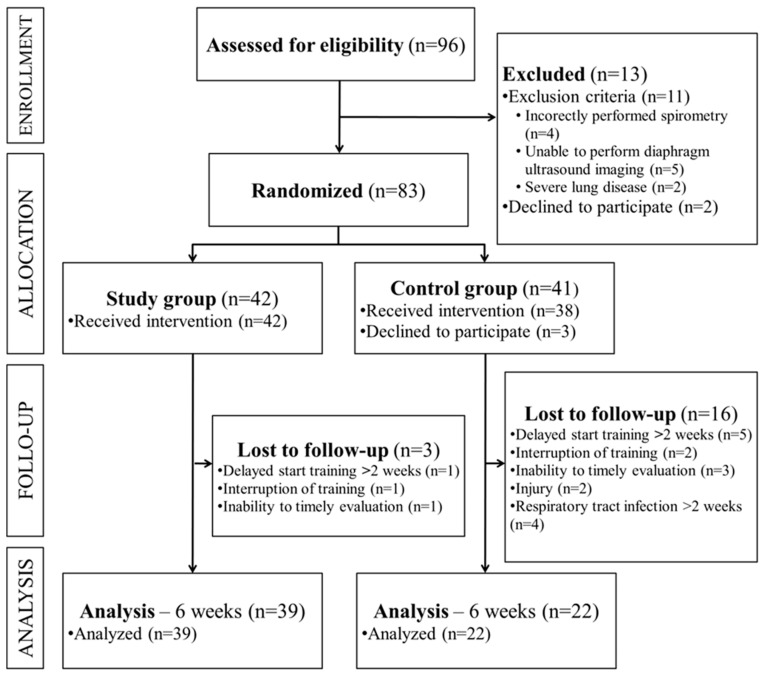
Diagram illustrating the flow of participants through the study.

After randomization, not all participants qualified for the completion of the rehabilitation project. The large majority of excluded participants were those from the control group, as shown in the flow diagram (Figure 1). The main problem was the delay in training sessions. If the participant delayed training more than 2 weeks, the rehabilitation result could be disproportionate, especially in the case of inspiratory muscle training (IMT) and the load progression used in it. Patients included in the analysis were those with adherence above 12 out of the planned 18 training sessions.

A total of 59 patients (37 women and 22 men) with a history of COVID-19 were included in the study. The mean age was 63.1 ± 13.41 years (range: 27 to 86 years). Details of the characteristics of patients in each group are presented in Table 2. All 59 patients underwent and completed a 6-week rehabilitation program according to the guidelines and under the supervision of physiotherapists. During rehabilitation, the overall health of each patient did not deteriorate, and no significant or serious adverse events were reported. There were four cases of respiratory infections lasting longer than two weeks and two mild respiratory infections that did not affect training attendance. At the beginning of the training, 14 patients reported increased fatigue and dyspnea, which was disproportionate to their level of activity. Additionally, 3 patients experienced minor discomfort in the hip region, and 2 patients reported discomfort in the knee joints, which was consistent with their health and age.

We explored statistical relations and changes within all spirometry results and diaphragm mobility as well as muscle strength before and after assessed pulmonary rehabilitation. From all results we found significant changes in PImax, PEmax, FEV1%FVC, FEV1%VC MAX, FEV1, VC MAX, FVC EX, FVC IN, PEF, MEF25, DTF on both sides, diaphragm thickness during maximal inhalation, and chest expansion at xiphoid process and 10th rib levels between the two groups of patients—before and after rehabilitation (*p* < 0.05, confidence interval 95%). The median values with interquartile range (IQR) of parameters improved after pulmonary rehabilitation are presented in Table 3. 

**Figure 2:**
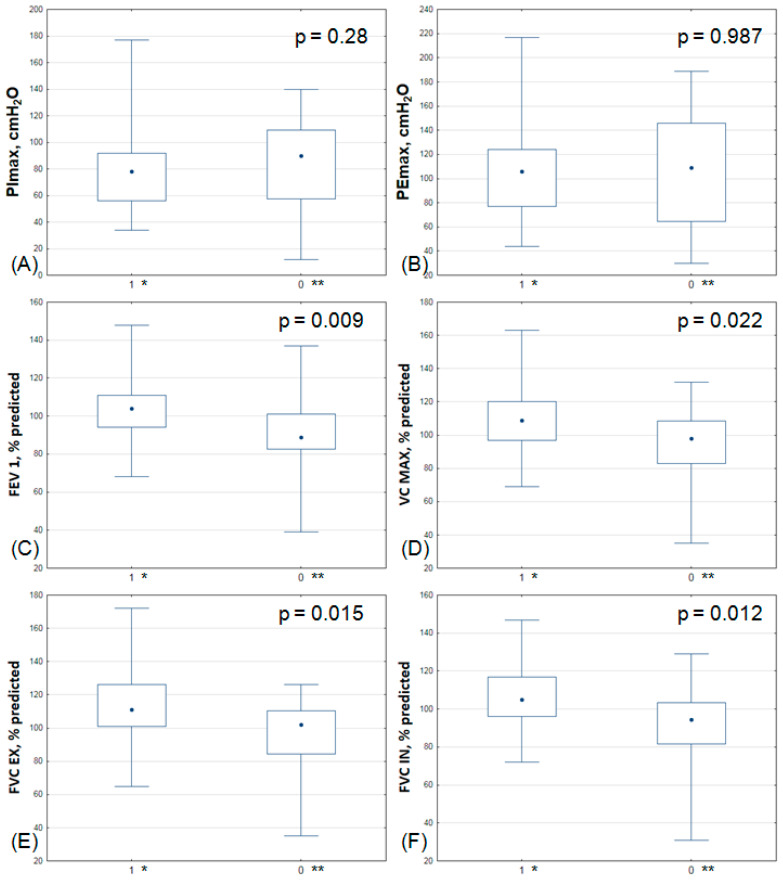
Comparison of the applied training regimens in the study and control groups showing primary outcomes and spirometric parameters. (**A**) PImax—maximal inspiratory pressure; (**B**) PEmax—maximal expiratory pressure; (**C**) FEV1—forced expiratory volume in 1 s; (**D**) VC MAX—maximum vital capacity; (**E**) FVC EX—forced expiratory vital capacity; (**F**) FVC IN—forced inspiratory vital capacity. The box represents the interquartile range between the 25th and 75th percentiles, where the median is denoted by a central dot, and the whiskers indicate the minimum and maximum values. 1 *: study group (IMT); 0 **: control group (placebo).

Comparisons between the intervention and control groups for all primary and secondary outcomes were conducted. The exact values of the selected parameters are presented in Figure 2 below. We observed that the majority of the analyzed functional parameters, including diaphragm changes, showed no significant differences in the post-rehabilitation group, as presented in Table 4.

Additionally, we found correlations (*p* < 0.05) between the below-stated parameters in the study group before treatment:

- ***PImax*** and: PEmax (r = 0.576), chest expansion at the level of xiphoid process (r = 0.484), chest expansion at the level of 10th rib (r = 0.408), PEF (r = 0.327), right hemidiaphragm thickness during maximal expiration (r = 0.339);
- ***PEmax*** and: PImax (r = 0.576), chest expansion at the level of 10th rib (r = 0.394).

In the control group:

- ***PImax*** and: PEmax (r = 0.554), FEV1 (r = 0.627), VC MAX (r = 0.584), FVC EX (r = 0.494), FVC IN (r = 0.595), PEF (r = 0.515), right hemidiaphragm thickness during maximal inspiration (r = 0.627), left hemidiaphragm thickness during maximal inspiration (r = 0.547);
- ***PEmax*** and: PImax (r = 0.554), FEV1 (r = 0.509), PEF (r = 0.46).

On the other hand, the most significant correlations in the study group after the treatment were between:

- ***PImax*** and: PEmax (r = 0.433), chest expansion at the level of xiphoid process (r = 0.319), chest expansion at the level of 10th rib (r = 0.322);
- ***PEmax*** and: PImax (r = 0.433), chest expansion at the level of 10th rib (r = 0.371).

In the control group:

- ***PImax*** and: PEmax (r = 0.729), PEF (r = 0.5) right hemidiaphragm thickness during maximal inspiration (r = 0.445);
- ***PEmax*** and: PImax (r = 0.729), PEF (r = 0.6) right hemidiaphragm thickness during maximal inspiration (r = 0.569).

In addition to PEF, none of the other spirometric parameters correlated with any of the other parameters measured after the intervention.

## 4. Discussion

In our study, a comprehensive medical and physiotherapeutic assessment was performed to personalize the rehabilitation program and evaluate lung function parameters in post-COVID-19 patients. Our data suggest that usage of pulmonary rehabilitation improves chest expansion, broadens vital and forced capacity of the lungs and movement of the diaphragm, and improves expiration ability. As the correlations between specific parameters remained consistent, the differences in r and *p*-values suggest that rehabilitation, as a modulating factor, impacted not only the parameter values but also their relationships. The correlations mentioned above provide a wide range of possibilities for further research into the mutual dependencies between chest expansion parameters and PImax and PEmax.

The rehabilitation program designed for this study resulted in an enhancement of PImax and PEmax following the intervention period compared with baseline values. The results after our patients’ rehabilitation indicate a significant improvement in respiratory muscle strength, much greater than those reported in earlier available studies [44,45,46]. We observed a higher average increase in PImax values in the control group compared with the study group. This difference may be attributed to variations in final group sizes, as well as patients’ mental and physical health levels. Moreover, our rehabilitation program included not only respiratory muscle training but also aerobic, strengthening, and stretching exercises. Perhaps incorporating manual therapy, especially for the chest and diaphragm, could lead to even greater improvements in respiratory muscle strength, and consequently, effective and long-lasting reduction in dyspnea and fatigue symptoms. We hope that this difference in group size will not be a limitation in subsequent analyzes as we continue to qualify participants. Such a large increase in the PImax value shows that COVID-19 has a long-term negative impact on the strength of the respiratory muscles, especially the inspiratory muscles.

To the best of our knowledge, this is the first report comparing changes in chest mobility among patients with post-COVID-19 syndrome. The applied rehabilitation program led to an increase in chest mobility at both levels, with improvement observed in both groups. Greater improvement was achieved in the study group, with an improvement of 68.93% in the upper measurement and 40.91% in the lower measurement. In the control group, these results were 64.9% and −7.45%, respectively. The significant difference may be attributed to the different group sizes, but only in the intervention group were the results statistically significant, suggesting a higher effectiveness of pulmonary rehabilitation using the trainer. Lanza et al. explain the relationship between chest expansion and respiratory muscle strength using the example of healthy subjects [47]. Larger axillary and thoracic circumferential measurements are linked to higher maximum inspiratory and expiratory pressure, FEV1, FVC, and inspiratory capacity. It should be noted, however, that age and BMI may influence chest expansion [48]. We did not find sufficiently strong correlations between spirometric parameters and chest wall expansion measurements. Only in the study group, there was a moderate correlation between chest measurement at the level of xiphoid process and FVC EX and VC MAX, which supports the above thesis.

Almost all spirometric parameters improved after rehabilitation in both the study group and the control group. Nopp et al. conducted a prospective observational cohort study that included consecutive patients admitted to an outpatient pulmonary rehabilitation center due to persistent symptoms after COVID-19. Following 6-week rehabilitation, individuals with long COVID-19 exhibited improvement in their quality of life, reduced dyspnea, decreased fatigue, enhanced exercise capacity, and a 6.9% increase in FEV1 (*p* = 0.011) [49]. Previous observations have already indicated that in the early phase of COVID-19 illness, abnormalities in FEV1, FVC, and the small airways are present [27]. However, follow-up studies in adults suggest that spirometry results are normal or close to normal [50,51,52]. Hence, the results are inconsistent, and the long-term effects of COVID-19 on individuals still require determination. Despite the presence of persistent respiratory symptoms during qualification, including exertional and non-exertional dyspnea, the average spirometric parameters for all patients were as follows: FEV1—98% in study group, 88% in control; FVC EX—103% in study group, 89% in control. Notably, the applied rehabilitation even improved these relatively high parameters. This may suggest a positive influence of respiratory muscle strength on the overall respiratory system condition and the patients’ efficiency.

Following a six-week rehabilitation program, an ultrasound examination revealed an augmented thickness of both the right and left hemidiaphragms during maximum inspiration. Notably, only the left hemidiaphragm revealed increased thickness during a regular inspiration. As the first measurement of normal breathing was on the right side, it is possible that the participant had greater respiratory control when measuring it on the left side. Additionally, there was an observed increased DFT on both sides after rehabilitation. Veldman et al. conducted a prospective cohort study with a 1-year follow-up to assess diaphragm function using ultrasonography. They affirmed the existence of diaphragm dysfunction during COVID-19 hospitalization. This confirmation was based on abnormal values detected in the hospital and further supported by notable improvements observed over the span of one year. The estimated mean DTF showed a progression from 0.56 at the time of admission to 0.78 at discharge or within 7 days of admission. It further increased to 1.05 at 3 months after admission and eventually reached 1.54 at 12 months after admission [53]. Prolonged hospitalization or home self-isolation for COVID-19 patients, as well as myopathy induced by the presence of the SARS-CoV-2 virus, can lead to muscle deconditioning, including the diaphragm [52]. Weakening of the diaphragm and its extended recovery can accelerate comprehensive rehabilitation. We obtained the greatest increase in diaphragm thickness during its maximum contraction. Moreover, we observed a relatively greater increase in the group in which inspiratory muscle training was included in the rehabilitation. Various studies have demonstrated the multidimensionality of diaphragm function in post-COVID-19 patients. It has been shown that greater improvements in respiratory function and diaphragm thickness were achieved when utilizing thoracic mobilization, lumbar stabilization exercises, and lower limb ergometer exercises compared with a combination of breathing exercises and lower limb ergometer exercises [22,54]. 

As our studies have demonstrated a positive impact on the respiratory system, further comprehensive research on the effectiveness of the program is necessary. Subsequent investigations should explore the enduring effects of the rehabilitation program over an extended period. Assessing patients beyond the immediate post-rehabilitation phase would provide valuable insights into the program’s sustainability and its ability to maintain positive outcomes over time. Conducting a comparative analysis with other existing rehabilitation programs could offer a deeper understanding of the distinct advantages and limitations of the presented intervention. Identifying best practices and potential areas for improvement could guide the refinement of rehabilitation strategies. Future research should explore how the presence of comorbidities influences the effectiveness of the rehabilitation program. This could involve stratifying participants based on specific health conditions to tailor interventions more precisely. In light of the evolving healthcare system, it is crucial to explore the effectiveness of remotely delivered rehabilitation programs. Examining telerehabilitation or digital platforms as alternative modes of program delivery could improve accessibility and cater to the diverse needs of patients. A deeper exploration of the impact of lifestyle modifications and behavioral changes on rehabilitation outcomes is warranted. Understanding how factors such as physical activity, dietary habits, and mental health contribute to the program’s success would inform comprehensive patient care.

Integrating the components of the presented rehabilitation or amalgamating them with existing rehabilitation programs across diverse centers, may prove highly effective. This is particularly noteworthy as the proposed program does not necessitate costly or highly specialized equipment. Of significant value would be the incorporation of mandatory respiratory exercises, with a particular emphasis on strengthening respiratory muscles. Notably, in Poland, there is currently a lack of standardized and reimbursed programs specifically designed for individuals experiencing prolonged effects of COVID-19. Therefore, the implementation within various rehabilitation programs of additional methods aimed at enhancing the respiratory system could positively impact the health of post-COVID patients. Hence, it is crucial to broaden the scope of the study to include a more representative sample of patients whose health has shown significant improvement.

### 4.1. Generalisability 

Given that the intervention was administered across both sexes, diverse age groups, individuals experiencing varied trajectories of COVID-19, and with varying degrees of dyspnea and activity levels, the findings suggest potential benefits for patients who adopt the presented rehabilitation program as a preventive measure against long-term COVID-19 symptoms. Our study presents only a subset of results, highlighting the need for analyses based on a significantly broader dataset. We believe that expanding the dataset is crucial for gaining a more comprehensive understanding of the effectiveness of our rehabilitation program across diverse demographic groups.

### 4.2. Limitations

One should note a limitation of this study, which is an uneven number of participants subjected to the final analysis due to unforeseen random events. Moreover, in the current analysis, we did not demonstrate the impact of a range of factors that could potentially influence the outcomes of the pulmonary rehabilitation program. These factors include comorbidities such as cardiovascular disease or diabetes, the severity and duration of COVID-19 symptoms, medication usage that might affect respiratory or muscular function, lifestyle factors such as physical activity levels and diet, psychological factors including mental health status, and socioeconomic factors like income and access to healthcare resources. It is important to emphasize that the data presented here constitute a portion of an ongoing larger project registered with ClinicalTrials.gov (NCT05449379).

## 5. Conclusions

The applied rehabilitation program, which included aerobic training on a cycle ergometer, respiratory training, resistance training, overall fitness exercises, and stretching routines, is highly effective in improving the functional parameters of the respiratory system. The primary outcome showed that the use of additional resistance training devices is not necessary to achieve satisfactory results. The high effectiveness of the intervention allows for the development of a high-quality rehabilitation protocol. However, there is still a need to assess various training programs to find the most effective rehabilitation program for post-COVID-19 patients.

## Figures and Tables

**Table 1:** Progression of training load during the 6-week intervention.

Week	% HRmax or % PImax
1	45–55
2	50–60
3	55–65
4	60–70
5	65–75
6	70–80

HRmax: maximum heart rate; PImax: maximum inspiratory pressure.

**Table 2:** Patient characteristics after random assignment to study group and control group. The mean values of the results are presented along with the standard deviation.

	Study (IMT) Group	Control (Placebo) Group	*p*-Value
patients, *n*	39	22	
mean age, years	65.41 ± 11.23	57.90 ± 16.02	0.057
Male sex, % (*n*)	33 (13)	40 (9)	
Female sex, % (*n*)	67 (26)	60 (13)	
Weight, kg	78.74 ± 13.15	84.49 ± 23.07	0.548
Height, cm	167.49 ± 8.75	170.40 ± 8.15	0.181
Body mass index, kg/m^2^	28.01 ± 3.63	28.77 ± 6.19	0.701
History of smoking, % (*n*)	10 (4)	23 (5)	
PImax, cmH_2_O	51.62 ± 23.96	54.45 ± 34.19	0.943
PEmax, cmH_2_O	66.95 ± 25.65	73.55 ± 36.03	0.481
FEV1, % predicted	97.67 ± 20.33	87.65 ± 21.83	0.078
FVC EX, % predicted	103.38 ± 23.95	88.95 ± 17.72	0.016
FVC IN, % predicted	96.41 ± 31.05	71.85 ± 29.96	0.005
PEF, % predicted	93.56 ± 18.92	93.75 ± 29.52	0.854
VC MAX, % predicted	101.21 ± 23.39	87.05 ± 19.50	0.028
Xiphoid process level of chest expansion, cm	2.23 ± 1.53	3.05 ± 2.61	0.6
10th rib level of chest expansion, cm	1.32 ± 1.94	1.99 ± 1.89	0.09
DTF right side	1.345 ± 1.226	1.519 ± 1.314	0.325
DTF left side	1.375 ± 0.927	1.118 ± 0.802	0.181

DTF: diaphragm thickening fraction; FEV1: forced expiratory volume in 1 s; FVC EX: forced expiratory vital capacity; FVC IN: forced inspiratory vital capacity; PEmax: maximum expiratory pressure; PEF: peak expiratory flow; PImax: maximum inspiratory pressure; VC MAX: maximum vital capacity.

**Table 3:** Effects of applied rehabilitation. The median values of the results are presented along with the interquartile range.

Measure		Group	Baseline	Post-Intervention	Change Mdn (%)	Change M (%)	*p*-Value
***Respiratory pressure***							
PImax, cmH_2_O		Study	**52 (31–70)**	**78 (56–92)**	**52**	**72.50**	**<0.001**
		Control	**50.5 (23.5–84.5)**	**90 (57.5–109)**	**47.54**	**118.66**	**<0.001**
PEmax, cmH_2_O		Study	**61 (48–83)**	**106 (77–124)**	**59.72**	**72.51**	**<0.001**
		Control	**66 (51–98.5)**	**109 (64.5–146)**	**37.9**	**62.23**	**<0.001**
***Chest expansion***							
Xiphoid process level, cm		Study	**2 (1–3)**	**3 (1.5–4.5)**	**66.67**	**68.93**	**0.001**
		Control	2.5 (0.5–5.5)	3 (1.75–3.75)	20.83	64.9	0.24
10th rib level, cm		Study	**1 (0–2)**	**2.5 (1–4.5)**	**66.67**	**40.91**	**0.001**
		Control	1.75 (0.75–3.25)	1.5 (1–4)	−5	−7.45	0.435
***Spirometry***							
FEV 1, % predicted		Study	**95 (86–108)**	**104 (94–111)**	**5.26**	**8.02**	**<0.001**
		Control	86.5 (75–100)	89 (82.5–101)	0.85	6.42	0.457
FVC EX, % predicted		Study	**103 (87–117)**	**111 (101–126)**	**4.07**	**13.24**	**<0.001**
		Control	84.5 (76.5–105)	102 (84.5–110.5)	0.87	9.69	0.205
FVC IN, % predicted		Study	105 (85–115)	105 (96–117)	1.89	34.82	0.07
		Control	**75.5 (42–100)**	**94.5 (81.5–103.5)**	**8.18**	**53.96**	**0.023**
VC MAX, % predicted		Study	**102 (89–114)**	**109 (97–120)**	**4.35**	**12.5**	**0.002**
		Control	80 (73.5–105.5)	98 (83–108.5)	0	10.5	0.156
FEV1%FVC EX, % predicted		Study	101 (95–109)	100 (95–103)	−1.89	−2.48	0.088
		Control	**110 (103–123)**	**103.5 (95.5–110)**	**−5.24**	**−7.06**	**<0.001**
FEV1%VC MAX, % predicted		Study	98 (93–108)	99 (95–103)	−0.98	−2.19	0.172
		Control	**108 (101.5–111.5)**	**101 (96–108)**	**−3.94**	**−5.41**	**0.002**
PEF, % predicted		Study	**95 (81–108)**	**102 (88–113)**	**8.86**	**9.67**	**<0.001**
		Control	97.5 (69–120.5)	96.5 (78–110.5)	4.63	9.97	0.542
MEF 75, % predicted		Study	91 (78–105)	89 (75–113)	4.62	4.44	0.341
		Control	83.5 (73.5–117)	89.5 (80–104.5)	−7.21	4.89	0.059
MEF 50, % predicted		Study	76 (56–89)	76 (57–87)	0	−3.34	0.478
		Control	**82.5 (63.5–102)**	**79 (62–101.5)**	**−9.2**	**−4.19**	**0.044**
MEF 25, % predicted		Study	70 (50–84)	61 (46–76)	−2.06	−0.21	0.16
		Control	**83 (58–126)**	**68 (56.5–86.5)**	**−17.65**	**−16.26**	**0.026**
***Diaphragm ultrasonography***							
Maximum inhalation, cm	Right hemidiaphragm	Study	**0.415 (0.321–0.477)**	**0.486 (0.401–0.556)**	**15.25**	**22.75**	**<0.001**
		Control	**0.413 (0.351–0.485)**	**0.448 (0.378–0.561)**	**5.62**	**12.61**	**<0.001**
	Left hemidiaphragm	Study	**0.417 (0.359–0.483)**	**0.465 (0.387–0.586)**	**14.91**	**18.64**	**<0.001**
		Control	**0.407 (0.354–0.488)**	**0.429 (0.356–0.526)**	**3.31**	**7.53**	**0.002**
Maximum exhalation, cm	Right hemidiaphragm	Study	0.201 (0.162–0.244)	0.17 (0.132–0.247)	−13.53	18.1	0.645
		Control	0.174 (0.139–0.208)	0.174 (0.147–0.201)	−7.84	2.42	0.681
	Left hemidiaphragm	Study	0.18 (0.157–0.211)	0.185 (0.154–0.225)	0	9.71	0.561
		Control	0.19 (0.17–0.256)	0.175 (0.156–0.224)	−5.44	−7.27	0.087
Normal inhalation, cm	Right hemidiaphragm	Study	0.254 (0.201–0.285)	0.232 (0.193–0.355)	3.78	40.41	0.18
		Control	0.212 (0.171–0.278)	0.216 (0.190–0.286)	7.12	9.27	0.433
	Left hemidiaphragm	Study	**0.204 (0.186–0.24)**	**0.217 (0.193–0.301)**	**11.76**	**20.1**	**0.003**
		Control	0.21 (0.198–0.267)	0.221 (0.195–0.294)	1.48	9.04	0.094
Normal exhalation, cm	Right hemidiaphragm	Study	0.201 (0.162–0.244)	0.216 (0.154–0.27)	3.02	28.58	0.376
		Control	0.175 (0.139–0.208)	0.178 (0.145–0.213)	−7.84	4.38	0.94
	Left hemidiaphragm	Study	0.18 (0.157–0.211)	0.185 (0.154–0.225)	0	9.53	0.544
		Control	0.19 (0.17–0.256)	0.172 (0.156–0.233)	−8.14	−6.43	0.064
DTF	Right hemidiaphragm	Study	**0.882 (0.625–1.594)**	**1.441 (0.863–2.384)**	**43.03**	**101.69**	**0.036**
		Control	1.034 (0.905–1.413)	1.085 (0.899–2.164)	37.51	50.9	0.313
	Left hemidiaphragm	Study	**1.232 (0.778–1.782)**	**1.418 (1.015–2.211)**	**26.08**	**40.92**	**0.021**
		Control	**0.902 (0.657–1.355)**	**1.15 (0.827–2.114)**	**18.38**	**142.98**	**0.008**

DTF: diaphragm thickening fraction; FEV1: forced expiratory volume in 1 s; FVC EX: forced expiratory vital capacity; FVC IN: forced inspiratory vital capacity; MEF (25–75%): maximal expiratory flow in the range 75–25% of FVC; PEmax: maximum expiratory pressure; PEF: peak expiratory flow; PImax: maximum inspiratory pressure; VC MAX: maximum vital capacity.

**Table 4:** Statistical comparisons between the intervention and control groups for all primary and secondary outcomes. The medians of the results along with the interquartile range and the effect size (r) were presented.

Measure		Change		r	*p*-Value
		Study	Control		
***Respiratory pressure***					
PImax, cmH_2_O		24 (8–33)	23.5 (9.5–34)	0.141	0.28
PEmax, cmH_2_O		35 (16–63)	18.5 (7.5–59)	0.002	0.987
***Chest expansion***					
Eiphoid process level, cm		1 (0–2)	0.5 (−0.45–1)	0.023	0.86
10th rib level, cm		1 (0–2.5)	0 (−0.5–1.5)	0.06	0.646
***Spirometry***					
**FEV 1, % predicted**		**5 (−1–9)**	**1 (−10–12)**	**0.338**	**0.009**
**FVC EX, % predicted**		**5 (0–11)**	**1 (−4.5–21.5)**	**0.317**	**0.015**
**FVC IN, % predicted**		**2 (−3–11)**	**9 (−0.5–42.5)**	**0.327**	**0.012**
**VC MAX, % predicted**		**4 (−2–9)**	**0 (−5–15.5)**	**0.299**	**0.022**
FEV1%FVC EX, % predicted		−2 (−6–3)	−5.5 (−12.5 to −2)	0.155	0.232
FEV1%VC MAX, % predicted		−1 (−5–3)	−4 (−10 to −1.5)	0.151	0.248
PEF, % predicted		8 (−2–15)	4.5 (−8.5–12.5)	0.135	0.301
MEF 75, % predicted		4 (−4–14)	−8.5 (−16–10)	0.071	0.586
MEF 50, % predicted		0 (−10–8)	−8 (−14.5–1)	0.093	0.476
MEF 25, % predicted		−2 (−20–8)	−11.5 (−28 to −2)	0.118	0.365
***Diaphragm ultrasonography***					
Maximum inhalation, cm	Right hemidiaphragm	−0.018 (−0.054–0.077)	0.023 (0.013–0.092)	0.056	0.665
	Left hemidiaphragm	0.062 (0.034–0.095)	0.013 (0.005–0.042)	0.115	0.378
Maximum exhalation, cm	Right hemidiaphragm	−0.018 (−0.054–0.077)	−0.015 (−0.034–0.031)	0.044	0.736
	Left hemidiaphragm	0 (−0.023–0.031)	−0.012 (−0.033–0.005)	0.038	0.773
Normal inhalation, cm	Right hemidiaphragm	0.009 (−0.07–0.116)	0.014 (−0.023–0.05)	0.145	0.266
	Left hemidiaphragm	0.023 (−0.006–0.085)	0.005 (−0.004–0.043)	0.021	0.873
Normal exhalation, cm	Right hemidiaphragm	0.005 (−0.054–0.092)	−0.015 (−0.038–0.046)	0.177	0.173
	Left hemidiaphragm	0 (−0.023–0.031)	−0.014 (−0.033–0.005)	0.04	0.761
DTF	Right hemidiaphragm	0.48 (−0.217–1.159)	0.24 (−0.517–0.78)	0.023	0.829
	Left hemidiaphragm	0.211 (−0.008–0.766)	0.266 (−0.003–0.598)	0.105	0.419

DTF: diaphragm thickening fraction; FEV1: forced expiratory volume in 1 s; FVC EX: forced expiratory vital capacity; FVC IN: forced inspiratory vital capacity; MEF (25–75%): maximal expiratory flow in the range 75–25% of FVC; PEmax: maximum expiratory pressure; PEF: peak expiratory flow; PImax: maximum inspiratory pressure; VC MAX: maximum vital capacity.

## Data Availability

Data supporting reported results can be provided upon request from the corresponding author.
